# Rapid Fabrication of MgNH_4_PO_4_·H_2_O/SrHPO_4_ Porous Composite Scaffolds with Improved Radiopacity via 3D Printing Process

**DOI:** 10.3390/biomedicines9091138

**Published:** 2021-09-02

**Authors:** Xiaofeng Cao, Wufei Ge, Yihu Wang, Ming Ma, Ying Wang, Bing Zhang, Jianing Wang, Yanchuan Guo

**Affiliations:** 1 Key Laboratory of Photochemical Conversion and Optoelectronic Material, Technical Institute of Physics and Chemistry, Chinese Academy of Sciences, Beijing 100190, China; xfcao@mail.ipc.ac.cn (X.C.); wyh8632@mail.ipc.ac.cn (Y.W.); maming@mail.ipc.ac.cn (M.M.); wangying@mail.ipc.ac.cn (Y.W.); zhangbing@mail.ipc.ac.cn (B.Z.); wangjianing@mail.ipc.ac.cn (J.W.); 2 Department of Orthopedics, The First Affiliated Hospital, Division of Life Sciences and Medicine, University of Science and Technology of China, Hefei 230022, China; gewufei@163.com; 3 School of Future Technology, University of Chinese Academy of Sciences, Beijing 100049, China

**Keywords:** magnesium phosphate, strontium hydrogen phosphate, 3D printing, scaffolds, radiopacity

## Abstract

Although bone repair scaffolds are required to possess high radiopacity to be distinguished from natural bone tissues in clinical applications, the intrinsic radiopacity of them is usually insufficient. For improving the radiopacity, combining X-ray contrast agents with bone repair scaffolds is an effective method. In the present research, MgNH_4_PO_4_·H_2_O/SrHPO_4_ 3D porous composite scaffolds with improved radiopacity were fabricated via the 3D printing technique. Here, SrHPO_4_ was firstly used as a radiopaque agent to improve the radiopacity of magnesium phosphate scaffolds. X-ray diffraction (XRD), scanning electron microscopy (SEM), and energy-dispersive spectroscopy (EDS) were used to characterize the phases, morphologies, and element compositions of the 3D porous composite scaffolds. The radiography image showed that greater SrHPO_4_ contents corresponded to higher radiopacity. When the SrHPO_4_ content reached 9.34%, the radiopacity of the composite scaffolds was equal to that of a 6.8 mm Al ladder. The porosity and in vitro degradation of the porous composite scaffolds were studied in detail. The results show that magnesium phosphate scaffolds with various Sr contents could sustainably degrade and release the Mg, Sr, and P elements during the experiment period of 28 days. In addition, the cytotoxicity on MC3T3-E1 osteoblast precursor cells was evaluated, and the results show that the porous composite scaffolds with a SrHPO_4_ content of 9.34% possessed superior cytocompatibility compared to that of the pure MgNH_4_PO_4_·H_2_O scaffolds when the extract concentration was 0.1 g/mL. Cell adhesion experiments showed that all of the scaffolds could support MC3T3-E1 cellular attachment well. This research indicates that MgNH_4_PO_4_·H_2_O/SrHPO_4_ porous composite scaffolds have potential applications in the bone repair fields.

## 1. Introduction

As an alternative to autologous and allogeneic bone grafts, artificial bone grafts have gained more and more attention in recent decades as they overcome the drawbacks of bone grafts and have shown great promise for bone tissue repair and regeneration [1,2,3,4,5,6]. Recently, the bone tissue engineering (BTE) strategy has been proved to possess enormous potential in repairing bone defects, and several synthetic materials, e.g., inorganic materials and polymers, are used to fabricate bone repair scaffolds, which are the essential part of the BTE strategy [7,8,9,10,11]. However, bone repair and regeneration are complex processes that include the structure reconstruction and functional recovery of new bones, which results in multifold requirements for bone repair scaffolds [12,13,14]. Furthermore, structural and functional requirements may be diverse as the clinical applications are different. However, scaffolds with a single component may not be able to satisfy the requirements completely for a given implant application [15,16,17,18]. In order to meet these clinical and structural requirements, composite scaffolds, through combining two or more components, are designed and fabricated carefully. The influence factors such as components, ratios, profiles, and microstructures need to be considered [16,17,18,19,20,21].

Commonly, bone repair scaffolds are required to possess high radiopacity for noninvasively tracking and imaging the filling effect in clinical operations, in vivo degradation, and new bone growth by X-ray-based techniques, such as fluoroscopy, computer tomography, and radiography [22,23,24,25,26,27,28,29,30,31,32]. However, the intrinsic radiopacity of bone repair scaffolds is usually insufficient, and it is difficult to distinguish them from natural bone tissues. For enhancing their radiopacity, X-ray contrast agents such as Ba-based [22,23], Zr-based [24,25,26], Bi-based [27,28,29], and Sr-based [30,31,32] compounds are added into them to construct the composite scaffolds. Although BaSO_4_ and ZrO_2_ are widely used in poly (methyl methacrylate) (PMMA) bone cements as the radiopacifier, there are potential physical, mechanical, and biological risks if the particles are released from the scaffolds as they are unabsorbable in the physiological environment [22,24,27]. Bismuth-based radiopaque agents such as Bi_2_O_3_, (BiO)_2_CO_3_, and Bi_2_(Al_2_O_4_)_3_ are insoluble too, and there are similar potential risks if they are released from the scaffolds [27,28,33,34]. Wu and co-workers [34] found that Bi_2_O_3_, the radiopacifier in calcium silicate bone cements, is harmful to cell proliferation, alkaline phosphatase (ALP) activity, and mineralization of human osteosarcoma MG63 cells. Recently, Sr-based radiopaque agents, especially the degradable species, have attracted more and more attention as they can simultaneously improve the radiopacity and bone repair capability of bone implants [30,31,32,34,35,36,37]. Various Sr-containing species such as SrCO_3_, SrX_2_, (X = F, Cl, Br, I), strontium ranelate (SrR), and strontium-substituted hydroxyapatite (SrHAp, maximum 100% Sr^2+^) have been evaluated and show great X-ray absorption when they are added into bone implants [30,31,32,34,35,36,37]. Furthermore, most of them are soluble (SrCl_2_ and SrBr_2_) or have relatively high solubility (SrCO_3_, SrHAp, and SrR) and can continuously release Sr^2+^, which is able to enhance osteoblast activity and inhibit osteoclast activity [38,39,40].

As a representative strontium phosphate species, strontium hydrogen phosphate (SrHPO_4_, DSPA) has been studied as a catalyst, proton conductor, surface conditioner, and environmental purifying agent [41,42,43]. In the bone repair field, it is considered as an ion exchanger biomaterial and commonly used as a Sr additive in calcium phosphate cements (CPCs), which resulted in prolonging the setting time, modification of the compressive strength, enhancement of proliferation and osteogenic differentiation of mouse osteoblast precursor cells (MC3T3-E1 cells), and an increase in material degradation [38,43,44]. However, it is quite rare to evaluate the radiopacity of SrHPO_4_ for bone repair tracking and imaging [38]. Carvalho and co-workers [35] evaluated the radiopacity of commercial dentin adhesives filled with Sr_10_(PO_4_)_6_(OH)_2_ as the X-ray contrast agent. SrHPO_4_ is merely the by-product of Sr_10_(PO_4_)_6_(OH)_2_ nanoparticles with minor contents in the preparation process. Except for this study, there is almost no research about the radiopaque application of SrHPO_4_ in the bone repair field.

Magnesium phosphate (MgP)-based biomaterials are a relatively new research field, which have cytocompatibility and non-toxicity, sustained dissolution and degradation in vitro/vivo, excellent adhesion to bones, and potential clinical applications, thus being considered as a promising alternative to calcium phosphates (CaP) [45,46,47,48,49,50,51,52]. Magnesium phosphates are very complex as they are a general term for a class of compounds which are composed of phosphate radicals, the magnesium ion, other ions, and various amounts of water molecules [48,53]. Similar to other synthetic bone grafts, the radiopacity of ceramic magnesium phosphates is insufficient, and they are hardly distinguishable from natural bone tissue [54]. Incorporation of Sr species could enhance the radiopacity of magnesium phosphate scaffolds. Meininger and co-workers [54] fabricated Sr-doped magnesium phosphate 3D porous scaffolds using Sr-substituted Mg_(3−x)_Sr_x_(PO_4_)_2_ powders as the raw materials. The experiment results showed that Sr substitution could significantly improve the radiopacity of magnesium phosphate scaffolds compared to that of pure struvite (MgNH_4_PO_4_·6H_2_O). However, high-temperature sintering and a following crush process were required to prepare the Sr-substituted Mg_(3−x)_Sr_x_(PO_4_)_2_ powders. Except for this study, there are few other reports on radiopacity research of MgP scaffolds.

As one of the most representative species, dittmarite (MgNH_4_PO_4_·H_2_O) is a rare mineral in nature and can be detected as the by-product in struvite-based magnesium phosphate cements [55,56,57]. Meanwhile, it is found as a component of urinary stones in some cases [58]. In previous studies, it was studied in the phosphorus recovery field and utilized as a recyclable fertilizer [59]. More recently, our group firstly fabricated ceramic dittmarite 3D porous scaffolds for potential bone repair applications [60]. In brief, dittmarite (MgNH_4_PO_4_·H_2_O) scaffolds are a relatively novel material in the bone repair field, and further studies are necessary to improve their performance to make them more suitable for clinical applications.

In this work, MgNH_4_PO_4_·H_2_O/SrHPO_4_ porous composite scaffolds were firstly prepared via the 3D printing process. SrHPO_4_ was mixed with MgO as the starting materials, and the final 3D porous composite scaffolds were fabricated by 3D printing combined with a hydrothermal cementation post-treatment. Here, SrHPO_4_ was firstly used as a radiopaque agent to enhance the radiopacity of the magnesium phosphate scaffolds. A facile homogeneous precipitation method was used to synthesize the SrHPO_4_ powder, and high-temperature sintering and the following crush processes were avoided. The physicochemical properties of the porous composite scaffolds, such as morphologies, components, sizes of pores and struts, internal structures, porosities, radiopacities, in vitro degradation, and release of the Mg, Sr, and P elements, were studied in detail. The cytotoxicity and cell affinity were evaluated as well.

## 2. Materials and Methods

### 2.1. Materials

Strontium acetate (Sr(CH_3_COO)_2_, AR, 99%) and urea (NH_2_CONH_2_, AR, 99%) were purchased from Aladdin (Shanghai, China). Ammonium dihydrogen phosphate (NH_4_H_2_PO_4_, AR, 99%), diammonium hydrogen phosphate ((NH_4_)_2_HPO_4_, AR, 99%), and potassium chloride (KCl, AR, 99.5%) were purchased from Sinopharm Chemical Reagent (Shanghai, China). Magnesium oxide (MgO, AR, 98%) was purchased from Jinke Fine Chemical Reagent (Tianjin, China). Ethylene Oxide/Propylene Oxide Block Copolymer (Pluronic F-127, BioReagent, M.W. ~12,600 g/mol) was purchased from Sigma-Aldrich (St. Louis, MO, USA). MC3T3-E1 osteoblast precursor cells and Alpha Modification Eagle Medium (α-MEM) were purchased from Chinese Academy of Medical Sciences (Beijing, China). Fetal bovine serum was purchased from Thermo Fisher Scientific Inc. (Waltham, MA, USA). PBS buffer (sterile, pH 7.4) was purchased from Solarbio (Beijing, China). All reagents were used without further purification.

### 2.2. Synthesis of SrHPO_4_ Powder

SrHPO_4_ powder was synthesized by a facile homogeneous precipitation process, employing Sr(CH_3_COO)_2_ as the Sr source and NH_4_H_2_PO_4_ as the P source. In a typical preparation procedure, Sr(CH_3_COO)_2_ (80 mmol) and NH_4_H_2_PO_4_/urea (80 mmol) were dissolved in 200 mL distilled water to form a clear and a transparent solution, respectively. A white precipitate was formed when Sr(CH_3_COO)_2_ solution was mixed with NH_4_H_2_PO_4_/urea solution at room temperature. The reactants were maintained in a water bath at 75 °C for 4 h with continuous stirring and then cooled to ca. 50 °C in air. The precipitate was washed by vacuum filter with deionized water three times. Then, the precipitate was dried in air at 60 °C for 24 h.

### 2.3. Fabrication of MgP/SrP 3D Porous Composite Scaffolds

**Figure 1:**
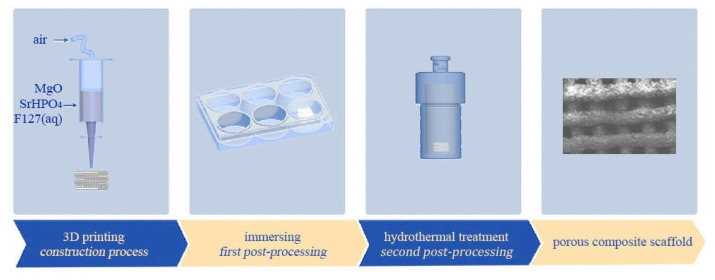
Schematic diagrams of manufacturing process of the MgNH_4_PO_4_·H_2_O/SrHPO_4_ porous composite scaffolds.

MgO and SrHPO_4_ powders without sintering were sieved with a 74 μm pore size mesh to avoid blocking the nozzle during the extruding procedure, and then they were mixed in a swing mixer (WAB AG, Switzerland) at various weight ratios, as listed in Table 1. The composite scaffolds with weight ratios of SrHPO_4_/MgO of 0:10, 1:9, 3:7, and 5:5 were recorded as S0, S1, S3, and S5, respectively. The parameters of cuboid porous scaffolds (base 10 × 10 mm, height 8 mm or 2 mm, porosity of 60%) with a 0°/90° alternating layer pattern were pre-designed and entered into a homemade 3D printing device equipped with a gas-pushed device. As shown in Figure 1, in the same manner as that of our previous report, the MgP/SrP 3D porous composite scaffolds were fabricated through a three-step process including (1) the 3D printing procedure in a 6-well plate, (2) the in situ immersion procedure after the 3D printing procedure, and (3) the hydrothermal procedure in a Teflon-lined stainless autoclave [60].

Briefly, in a batch fabrication process, 20 g of MgO/SrHPO_4_ powder was mixed with the binder of Pluronic F127 solution (0.15 g/mL, 15 mL) to form the printing “ink”. Subsequently, the paste was transferred into a syringe equipped a cylindrical nozzle with a 0.3 mm diameter. The green bodies with various sizes based on the pre-designed parameters were fabricated and dried at room temperature overnight and then in situ immersed in 0.5 mol/L of (NH_4_)_2_HPO_4_ solution for 1 d to obtain the primary scaffolds. In the following hydrothermal process, the primary scaffolds were transferred into a 50 mL Teflon-lined stainless autoclave containing a 30 mL mixture of NH_4_H_2_PO_4_-KCl-H_2_O, each with the concentration of 2 mol/L. The autoclave was sealed and heated at 120 °C for 2 h and then cooled to room temperature naturally. The porous scaffolds were washed and dried in air at 60 °C for 24 h.

### 2.4. Characterization of the Products

The phases of the products were characterized by powder X-ray diffraction using a D8 Focus diffractometer from Bruker with Cu K_α_ radiation (λ = 1.5406 Å) and recorded in the 2θ range of 5° to 70°. Scanning electron microscopy (SEM) and energy-dispersive spectroscopy (EDS) were performed on an FEG 250 scanning electron microanalyzer from Quanta with an operating voltage of 10 kV or 15 kV, and the samples were sputtered with Au plating to improve the electrical conductivity before the SEM observation. The Mg and Sr contents of the composite scaffolds were measured by a 710-OES inductively coupled plasma atomic emission spectrometer from Varian.

### 2.5. Porosity Measurement

Similar to our previous report [60], the porosity of the porous composite scaffolds (10 × 10 × 8 mm) was measured in a specific gravity bottle based on Archimedes’ principle. The calculation formula of the porosity is

Porosity (%) = [(W_2_ − W_3_ − W_s_)/ρ_e_]/[(W_1_ − W_3_)/ρ_e_] × 100%, (1)

where W_s_ is the weight of the scaffold, W_1_ is the weight of the specific gravity bottle filled with ethanol, W_2_ is the weight of the specific gravity bottle with ethanol and the scaffold, W_3_ is the weight of the specific gravity bottle with the rest of the ethanol and without the scaffold from W_2_, and ρ_e_ is the density of ethanol. Six specimens were tested for each scaffold.

### 2.6. X-ray Radiopacity

The radiopacity of the porous composite scaffolds was measured by a clinical Kodak DR7500 X-ray apparatus with an operating voltage of 65 kV and a current of 76 mA, and the quantitative analysis was conducted with the Computed Tomography software. An aluminum ladder (scaled 0.5–9 mm, with a height difference of 0.5 mm for every two adjacent steps) was used as the reference, and a standard curve (light vs. mm Al) was measured to determine the relative radiopacity of the porous scaffolds. Five specimens were tested for each scaffold (10 × 10 × 8 mm).

### 2.7. Degradation In Vitro

To measure the weight loss and ion release of the scaffolds, the porous composite scaffolds (10 × 10 × 2 mm) were separately soaked in Tris-HCl solution (pH 7.4, 0.05 M) with a volume-to-mass ratio of 200 mL/g in a bath shaker at 37 °C and 60 r/min for 1, 2, 3, and 4 weeks. At each time point, the residue weight rate was calculated based on the equation

Weight residue (%) = W_t_/W_0_ × 100%, (2)

where W_0_ is the initial weight of the scaffold, and W_t_ is the weight of the scaffold at the set time point. The Tris-HCl solution was refreshed, and the pH was measured at each time point. The accumulated concentrations of magnesium, strontium, and phosphorus released from the porous composite scaffolds were measured by inductively coupled plasma atomic emission spectrometry (ICP-AES). Four specimens were tested for each scaffold.

### 2.8. In Vitro Cytotoxicity Assay

The MTT test, an indirect method, was used to evaluate the in vitro cytotoxicity of the scaffolds. The porous composite scaffolds (10 × 10 × 8 mm) were sterilized by immersion in ethanol (75%) and simultaneous UV irradiation for 1 h at room temperature. After being washed in sterilized PBS solution three times, the porous composite scaffolds were immersed in α-modified essential media (α-MEM) supplemented with 10% fetal bovine serum (FBS) at 37 °C for 3 d, with the mass to volume of 0.1 g/mL. Considering the effect of the released ion concentrations on the in vitro cytotoxicity, the extracts with low concentrations of 0.05 and 0.01 g/mL were prepared by diluting the origin extracts with α-MEM supplemented with 10% FBS. MC3T3-E1 osteoblast precursor cells were seeded into 96-well plates at a density of 1 × 10^3^ cells per well and grown in α-MEM supplemented with 10% FBS under standard culture conditions. Six wells per treatment were prepared. After 24 h, non-attached cells were removed, and the media were replaced with 200 μL extracts with various concentrations of 0.1 g/mL, 0.05 g/mL, and 0.01 g/mL. α-MEM supplemented with 10% FBS was used as a blank control. Cells were cultured for 1, 3, and 5 d, followed by MTT cell viability assay measured at 490 nm on a microplate reader. The reported values were corrected by removing the interference of free Mg^2+^ [61].

### 2.9. Cell Adhesion on the Scaffolds

MC3T3-E1 osteoblast precursor cells were seeded onto the porous composite scaffolds (10 × 10 × 2 mm) with a density of 5 × 10^4^ cells in 24-well plates. After culturing for 3 and 5 d, cell/scaffold samples were washed thrice with PBS and fixed with 2.5% glutaraldehyde solution at 4 °C for 4 h. Cells were then dehydrated in ascending concentrations of ethanol (30, 50, 70, 90, 95, and 100% *v*/*v*) and dried in vacuum at 50 °C for 72 h. Finally, the cell/scaffold samples were observed on SEM.

### 2.10. Statistical Analysis

Statistical analysis was performed with SPSS software (v22, IBM, Armonk, NY, USA), using one-way ANOVA analysis, followed by Tukey post hoc tests to determine significance. Values are expressed as the mean ± standard deviation, and statistical significance was set at *p* < 0.05.

## 3. Results

### 3.1. Phases, Components, Morphologies, and Porosities of the Scaffolds

**Figure 2:**
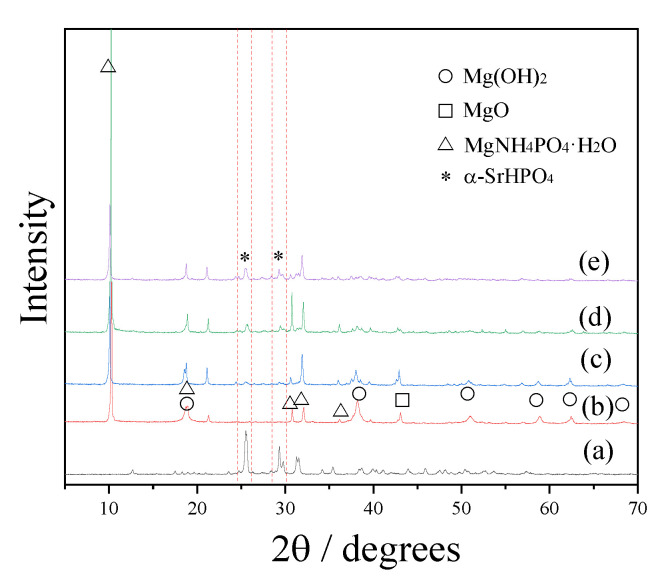
XRD patterns of α-SrHPO4 (**a**) and porous composite scaffolds S0, S1, S3, and S5 ((**b**–**e**), respectively), with increasing SrHPO_4_ contents. The diffraction peaks of α-SrHPO_4_ are marked by the red dotted lines.

Figure 2 shows the XRD patterns of the SrHPO_4_ powder and porous composite scaffolds with various SrHPO_4_ contents. Pattern (a) displays strong diffraction peaks that can be indexed to the anorthic phase of α-SrHPO_4_ (JCPDS 70-1215). XRD pattern (b) belongs to the S0 scaffolds, which is in agreement with our previous report that the components of the scaffolds are the orthorhombic phase of MgNH_4_PO_4_∙H_2_O (dittmarite, JCPDS 20-0663), hexagonal phase of Mg(OH)_2_ (JCPDS 07-0239), and cubic phase of MgO (JCPDS 89-4248) [60]. After adding α-SrHPO_4_ to the formulations of the composite scaffolds with various contents, the phases of the magnesium phosphate species were still MgNH_4_PO_4_∙H_2_O (patterns c, d, and e). Corresponding to the increased contents of SrHPO_4_, the diffraction peak intensities of SrHPO_4_ increase gradually. Meanwhile, the diffraction peaks indexed to Mg(OH)_2_ and MgO become weakened gradually. Based on the ICP-AES results, the Sr/Mg ratios of the porous composite scaffolds are close to the formulas (Table 1).

**Figure 3:**
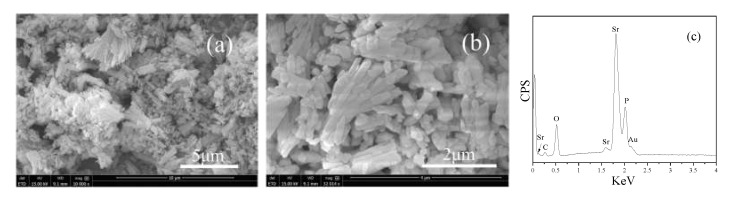
(**a**,**b**) SEM images and (**c**) EDS of α-SrHPO_4_.

Figure 3 shows the morphologies and element compositions of SrHPO_4_, indicating that SrHPO_4_ with a micro-scale size is the aggregate of the nanoplates, which is in agreement with a previous report [62]. The EDS data show the peaks belonged to Sr, P, and O. No other impurity peaks were detected, except the Au peak which can be attributed to the gold plating for increasing the electrical conductivity in the SEM measurements. Semi-quantitative analysis by the EDS spectrum shows that the molar ratio of Sr to P is ca. 1.08: 1, which is very close to the SrHPO_4_ stoichiometry.

**Figure 4:**
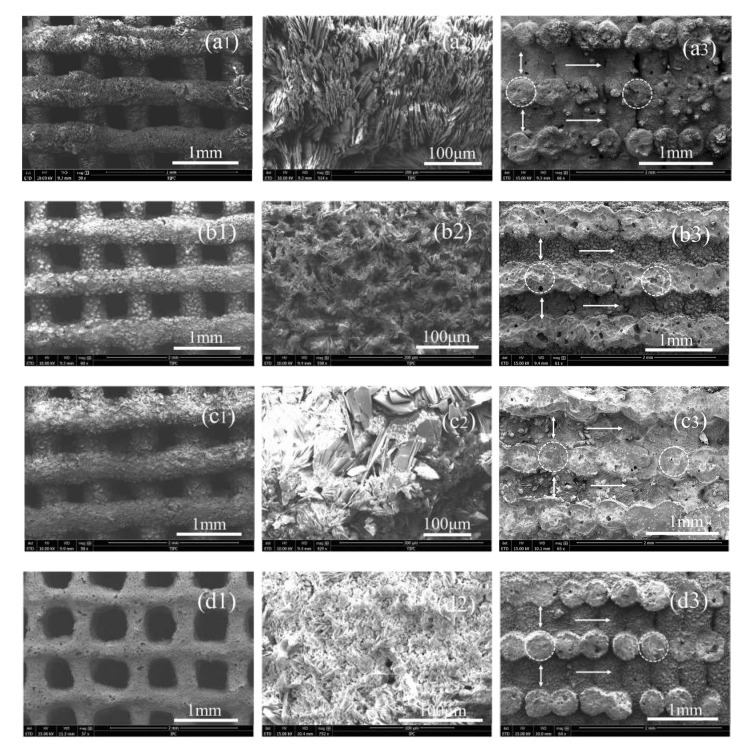
SEM images of porous composite scaffolds S0, S1, S3, and S5 ((**a**–**d**), respectively), with low-magnification SEM images (**a1**,**b1**,**c1**,**d1**), high-magnification SEM images (**a2**,**b2**,**c2**,**d2**), and internal structures (**a3**,**b3**,**c3**,**d3**).

The microstructure of the porous scaffolds was investigated by SEM (Figure 4), and the physical parameters of the surface strut diameters, pore sizes, internal tunnel sizes, and cross-section sizes of the struts are summarized in Table 2. As shown in Figure 4, the strut surface of all scaffolds is coarse, and pores with millimeter-scale sizes are clearly observed. The shaking during the extruding process causes the slight deviation in the size. The sizes are similar to each other in the S0, S1, and S3 scaffolds. The S5 scaffolds with a high content of SrHPO_4_ have smaller surface strut diameters and corresponding bigger pore sizes. A possible reason is that the high contents of SrHPO_4_ destroyed the completeness of the magnesium phosphate solidified network structures, which resulted in a few of the SrHPO_4_ and MgP particles falling from the surface of the scaffold struts. We noticed that a small amount of small white crystals, which had fallen from the scaffolds, were deposited on the bottom of the Teflon-lined stainless autoclave after the hydrothermal treatment. The element mapping of Mg, P, and Sr on the surface of the scaffolds was measured by EDS (Figure S1). Mg and P signals were detected and shown to overlap with each other. With increased contents of SrHPO_4_, the signals of element Sr were displayed and enhanced. Additionally, as shown in Figure 4, the down-up connected tunnels inside of the scaffold (marked by horizontal arrows) are observed clearly.

**Figure 5:**
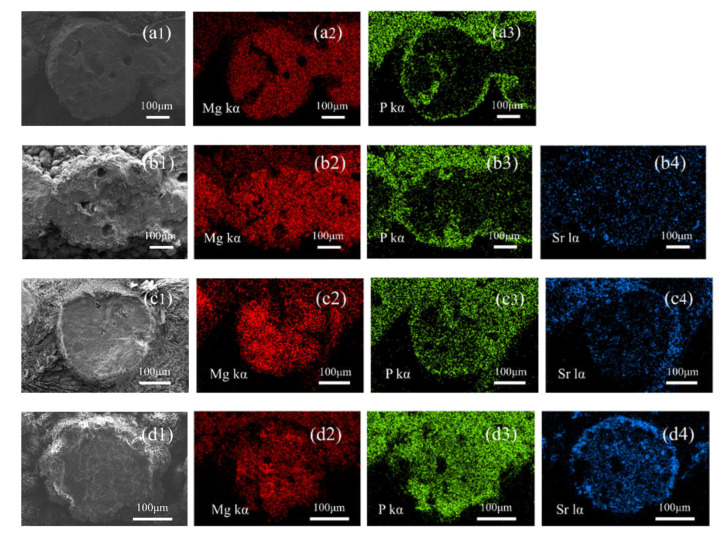
Cross-sectional morphologies and elemental mappings of porous composite scaffolds S0, S1, S3, and S5 ((**a**–**d**), respectively). SEM images (**a1**,**b1**,**c1**,**d1**); Mg element (**a2**,**b2**,**c2**,**d2**); P element (**a3**,**b3**,**c3**,**d3**); and Sr element (**b4**,**c4**,**d4**).

The inner Mg, P, and Sr distributions of the struts were also characterized by EDS elemental mapping. The (a) group in Figure 5 is the elemental mappings of the S0 scaffolds, which show that element Mg distributes inside and outside of the struts, and P mainly distributes at the edges of the struts. After adding SrHPO_4_ into the MgP scaffolds, element Mg is still the main component of the composite scaffolds. More element P is present in the further depths of the struts, and the signal intensities correspond to the contents of SrHPO_4_ (Figure 5). Similar to element Mg, element Sr distributes inside and outside of the struts, and with the increased contents of SrHPO_4_, the signal intensities of element Sr rise correspondingly (Figure 5 and Figure S1 ).

The porosities of the porous composite scaffolds are summarized in Table 1, which are lower than the setting ratio of 60%. The decrease in the porosities can be ascribed to the struts’ expansion when the paste was extruded from the nozzle and the layer spacing decreased, induced by the gravity of the scaffolds. The slightly higher porosity of S5 can be attributed to the slightly smaller diameter of the struts.

### 3.2. X-ray Radiopacity

**Figure 6:**
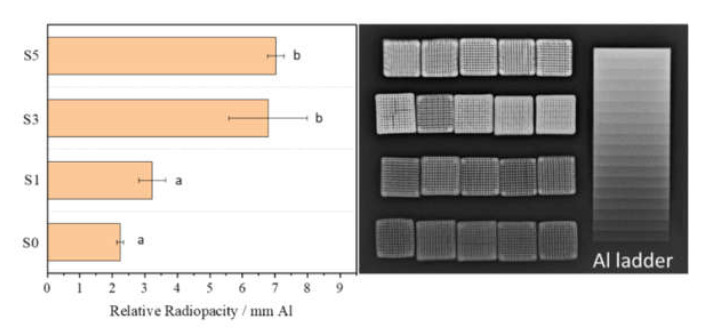
Relative radiopacity of MgNH_4_PO_4_·H_2_O/SrHPO_4_ porous composite scaffolds calculated by image analysis with respect to an aluminum standard. Different small letters represent statistically significant differences (*p* < 0.05), whereas the same small letters represent non-statistically significant differences (*p* > 0.05).

Figure 6 shows the radiopacity of the porous composite scaffolds, which indicates that with the increase in the SrHPO_4_ contents, the X-ray radiopacity of the composite scaffolds increases correspondingly. The S1 scaffolds with a SrHPO_4_ content of 2.42% have a similar radiopacity to the S0 scaffolds (*p* > 0.05), which have a relative X-ray radiopacity corresponding to 3.2 and 2.2 mm Al ladders, respectively. Further increasing the SrHPO_4_ contents to 9.34% and 21.79%, the S3 and S5 scaffolds have a similar radiopacity (*p* > 0.05), which are equal to 6.8 and 7.0 mm Al ladders, respectively.

### 3.3. In Vitro Degradation

**Figure 7:**
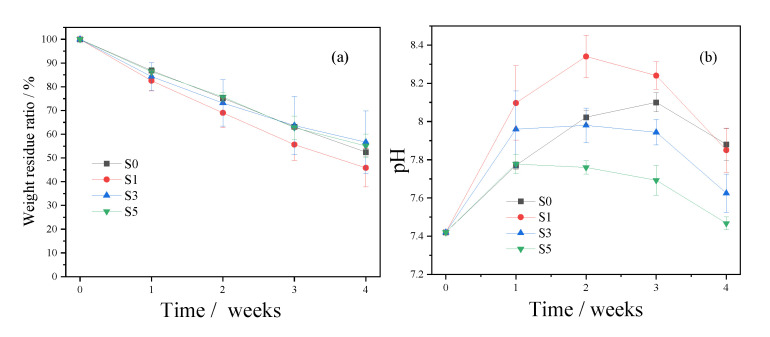
In vitro degradation of porous composite scaffolds. (**a**) Weight residue ratios of scaffolds; (**b**) pH values of Tris-HCl at the set time points.

**Figure 8:**
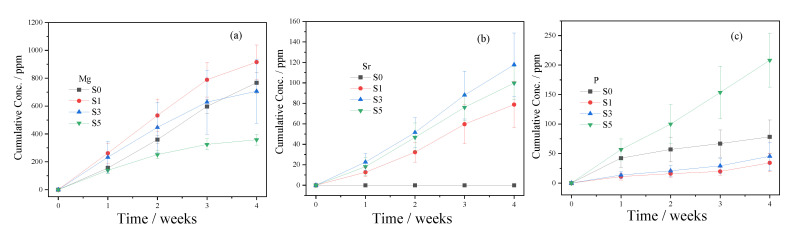
The accumulated concentration of Mg, Sr, and P elements ((**a**–**c**), respectively).

All of the scaffolds degraded gradually in vitro over 28 d, with weight residues of ca. 53%, 46%, 57%, and 55%, respectively, for S0, S1, S3, and S5 (Figure 7a). There were no statistically significant differences for each group (*p* > 0.05). The pH of the Tris-HCl solutions first increased to be slightly alkaline and then decreased (Figure 7b). The significant changes in the pH in the S1 group may be attributed to the fast degradation rate (Figure 7). The in vitro degradation of the scaffolds sustainably released the Mg, Sr, and P elements (Figure 8). It is noted that the degradation of the dittmarite porous scaffolds (S0 group) is faster than that of our previous research because the degradation media are different [60]. Here, Tris-HCl without the extra P element was used as the medium.

### 3.4. In Vitro Cytotoxicity Assay

**Figure 9:**
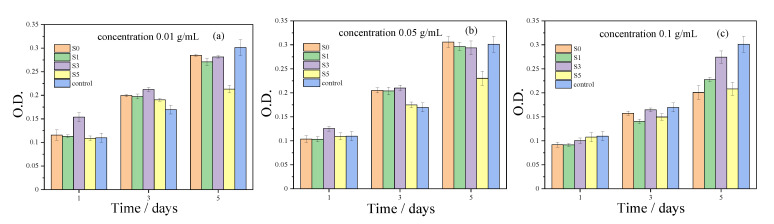
MTT cell viability assay of MC3T3-E1 osteoblast precursor cells exposed to various extract concentrations: (**a**) 0.01 g/mL, (**b**) 0.05 g/mL, and (**c**) 0.1 g/mL, for 1, 3, and 5 days.

The MTT assay showed that the MC3T3-E1 osteoblast precursor cells possess a viability of more than 70% in extracts of scaffolds with the concentrations of 0.01, 0.05, and 0.1 g/mL for 1 d, 3 d, and 5 d, compared with the control group (Figure 9). For the S5 scaffolds, the high SrHPO_4_ contents resulted in a decrease in cell proliferation.

**Figure 10:**
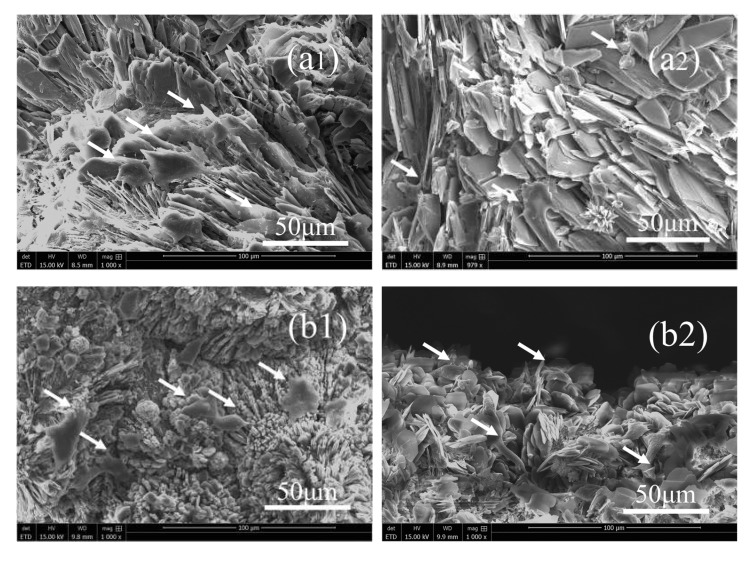

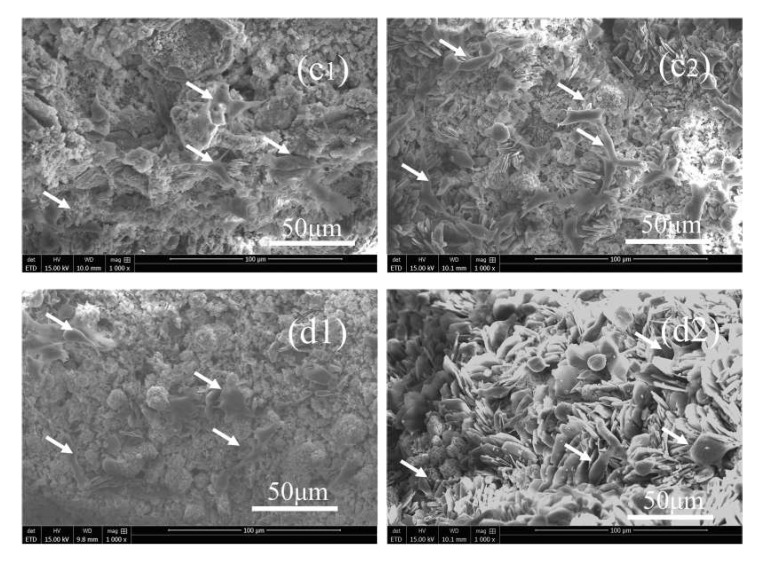
SEM images of the cell adhesion experiments of the porous composite scaffolds S0, S1, S3, and S5 (**a**–**d**) for 3 and 5 days. (**a1**,**b1**,**c1**,**d1**) for 3 days; (**a2**,**b2**,**c2**,**d2**) for 5 days.

The SEM images show that the MC3T3-E1 osteoblast precursor cells could attach and spread well on the composite scaffolds for 3d and 5d (Figure 10). The MTT test and cell adhesion results show that the S3 group with a SrHPO_4_ content of 9.34% possessed superior cytocompatibility compared to that of the pure MgNH_4_PO_4_·H_2_O scaffolds when the extract concentration was 0.1 g/mL, and all of the scaffolds had good cell affinity (Figure 9 and Figure 10).

## 4. Discussion

In the present research, novel MgNH_4_PO_4_·H_2_O/SrHPO_4_ porous composite scaffolds with improved radiopacity were firstly fabricated by a 3D printing process (Figure 1 and Figure 6). Magnesium phosphate-based bone graft materials are a relatively new field of research and are considered a promising alternative to calcium phosphate-based materials. Among them, dittmarite (MgNH_4_PO_4_·H_2_O) is a novel MgP material in the bone repair field and presents an enormous potential for bone repair [60].

Although magnesium phosphate (MgP)-based biomaterials possess excellent biocompatibility and bioactivity, the radiopacity of them is insufficient, making it difficult to distinguish them from natural bones [46,54]. In the present research, SrHPO_4_ was firstly used as a radiopaque agent to enhance the radiopacity of MgP porous scaffolds as Sr has a higher atomic weight than Mg and is an effective X-ray absorber. The SrHPO_4_ powder was prepared by a simple facile homogeneous precipitation process, and the MgO powder was a commercial light MgO, which avoided extreme high-temperature sintering and ball milling processes. Moreover, the light MgO powder without high-temperature treatment maintained a high chemical activity to make the scaffolds consolidate rapidly [60,63]. The MgO/SrHPO_4_ powder further mixed with the binder of Pluronic F127 solution to form an injectable paste, which possessed the self-setting property in a phosphate solution to fabricate the primary scaffolds at room temperature. The following hydrothermal process shortened the consolidation time to form the final scaffolds [60,63]. During the whole fabrication process, the cementation played an important role, and the sintering step at high temperature was not required. The X-ray radiopacity experiment results show that adding SrHPO_4_ significantly improved the radiopacity of the MgP porous scaffolds when the Sr/Mg reached 9.34% (Figure 6).

As bone repair materials, porous scaffolds are required to be cytocompatible and have minimal cytotoxicity [3]. Based on the previous reports, SrHPO_4_, which possesses good biocompatibility and an excellent bone repair effect, is a common additive to modify the physicochemical and biological properties of CaP biomaterials [38,43,44]. Additionally, our previous study showed that dittmarite (MgNH_4_PO_4_·H_2_O) 3D porous scaffolds had negligible cytotoxicity [60]. Therefore, it is rational to state that the MgNH_4_PO_4_·H_2_O/SrHPO_4_ porous composite scaffolds have good biocompatibility. Usually, in vitro cytotoxicity is related to the species released from the scaffolds, depending on the concentrations. Therefore, the extracts with various concentrations were evaluated to determine the in vitro cytotoxicity. As shown in Figure 9, the extracts of porous composite scaffolds with concentrations of 0.01, 0.05, and 0.1 g/mL had negligible cytotoxicity.

Additionally, cell adhesion to artificial scaffolds is fundamentally important for new bone formation and osseointegration in orthopedic surgery [64]. The SEM images showed that the MgNH_4_PO_4_·H_2_O/SrHPO_4_ porous composite scaffolds with various Sr contents could support MC3T3-E1 osteoblast precursor cells well, which proved that the composite scaffolds had good cell affinity for MC3T3-E1 osteoblast cells (Figure 10).

The pore size and interconnectivity are very important for osteoblast cell growth, vascularization, and diffusion of oxygen as well as nutrients into the scaffolds [3,65]. The previous reports proved that pores of scaffolds with the sizes of 0.1~0.4 mm in diameter are beneficial for osteoblast cell growth, and those 0.2~0.5 mm in diameter are beneficial for vascularization [3,65]. The degree of interconnectivity is often more important than the pore size for highly biodegradable porous ceramics as it can influence oxygen, nutrient, and waste exchange between the scaffolds and organisms [65]. As shown in Figure 4 and Table 2, the pore sizes of the composite scaffolds are close to the optimum size scopes, which is a benefit for osteoblast cell growth and/or vascularization. Additionally, the connected tunnels inside of the scaffolds are a benefit for cell ingrowth and matter exchange.

SrHPO_4_ did not influence the MgNH_4_PO_4_·H_2_O phase in the composite scaffolds and could stably exist inside and outside of the scaffold struts during the fabrication and the post-treatment processes (Figure 2, Figure 5, and Figure S1). Additionally, the SrHPO_4_ powder scarcely influenced the extrudability of the raw materials because of the small sizes and screening with the sample sieve (Figure 1 and Figure 3). SrHPO_4_ possesses a similar solubility to DCPA (CaHPO_4_) or DCPD (brushite, CaHPO_4_·2H_2_O), and DCPD is considered to have an ideal resorption rate in the body environment [38]. The in vitro degradation tests indicated that the MgNH_4_PO_4_·H_2_O/SrHPO_4_ porous composite scaffolds were sustainably degraded, and the Mg, Sr, and P elements were continuously released during the experiment periods, which resulted in the pH value changes of the Tris-HCl solutions (Figure 7 and Figure 8). The reasons could be ascribed to the hydration and dissolution processes of the porous composite scaffolds [63]. When the scaffolds were soaked in Tris-HCl solution, partial MgNH_4_PO_4_·H_2_O on the surface of the scaffolds degraded to form Mg^2+^, NH_4_^+^, and PO_4_^3−^. The inner MgO was exposed to the Tris-HCl solution and reacted with H_2_O to form Mg(OH)_2_. Abundant H_2_O promoted the dissolution of Mg(OH)_2_ to release Mg^2+^ and OH^−^, which induced the Tris-HCl system to be alkaline. When the immersion time was prolonged, the Mg(OH)_2_ coating on the surface of scaffolds delayed the hydration of MgO. Meanwhile, the dissolution of SrHPO_4_ could release Sr^2+^ and HPO_4_^2−^. The increase in the pH could result in the degradation of HPO_4_^2−^ to release H^+^. Partial OH^−^ was neutralized, and the pH value decreased. The release of Sr could enhance MC3T3-E1 cell proliferation (Figure 9). When the extract concentration was 0.1 g/mL, the MgNH_4_PO_4_·H_2_O/SrHPO_4_ porous composite scaffolds with a SrHPO_4_ content of 9.34% possessed enhanced cell proliferation compared to that of the pure MgNH_4_PO_4_·H_2_O scaffolds (Figure 9c). The possible reason was that Sr^2+^ could affect the calcium sensing receptor in a dose-dependent manner to increase the gene expression of c-fos and egr1, which are both involved in the regulation of osteoblast proliferation [66,67].

Here, further research is needed to characterize the in vitro performance of the porous composite scaffolds in the future. The mechanical properties and dynamic changes during the in vitro degradation are necessary for clinical application. Additionally, the cytotoxicity of the scaffold extracts with a wider range of concentrations should be evaluated, which will further reveal the relevance of the cytotoxicity and released ion concentrations. The osteogenic differentiation potential of the scaffolds needs to be evaluated, and the early-stage markers, such as Col I (collagen I) and ALP (alkaline phosphatase), and the terminal marker, such as OCN (osteocalcin) and OPN (osteopontin), should be characterized. Furthermore, biological safety studies such as genetic toxicity tests and inflammatory cytokine expression tests are required. These studies are the foundation for in vivo research.

In brief, novel MgNH_4_PO_4_·H_2_O/SrHPO_4_ porous composite scaffolds possess good biocompatibility, cell affinity, and enhanced radiopacity, which is suitable for noninvasive tracking of the implanting effect, scaffold degradation, and new bone ingrowth. The porous composite scaffolds have potential clinical applications in the orthopedic and craniofacial surgery fields to repair infected bone defects, accidental injury, tumorous bone defects, etc., through patient-customized routes. It is noted that the resorption time and hardness of the scaffolds must match the new bone growth.

## 5. Conclusions

In the present research, MgNH_4_PO_4_·H_2_O/SrHPO_4_ porous composite scaffolds with improved radiopacity were fabricated by a 3D printing process. SrHPO_4_ was firstly used as an X-ray contrast agent to improve the radiopacity of magnesium phosphate scaffolds. The radiography image proved that SrHPO_4_ was an effective X-ray absorber. When the SrHPO_4_ contents reached 9.34%, the radiopacity of the composite scaffolds was equal to that of a 6.8 mm Al ladder. The porous composite scaffolds exhibited good biocompatibility and cell affinity, sustained in vitro degradation, and suitable pore structures. This research shows the potential applications of MgNH_4_PO_4_·H_2_O/SrHPO_4_ porous composite scaffolds in the bone repair field.

## Figures and Tables

**Table 1:** The components and porosities of the MgNH_4_PO_4_·H_2_O/SrHPO_4_ composite scaffolds.

Scaffold	SrHPO4 wt%	MgO wt%	Sr:Mg ^a^	Sr:Mg ^b^	Porosity%
S0	0	100	0:100	0:100	50.85 ± 3.78%
S1	10	90	2.42:100	2.49:100	51.52 ± 4.77%
S3	30	70	9.34:100	9.06:100	50.29 ± 3.71%
S5	50	50	21.79:100	21.05:100	52.15 ± 3.73%

^a^ Calculation results of the reactant ratio, mol/mol. ^b^ Results of the ICP-AES test of the final scaffolds, mol/mol.

**Table 2:** The strut diameters, pore sizes, internal tunnel sizes, and cross-section sizes of the porous composite scaffolds.

Sample	Strut ^a^	Pore ^a^	Tunnel ^a^	Cross-Section ^a^
S0	0.35–0.52	0.28–0.45	0.30–0.45	0.35–0.48
S1	0.35–0.48	0.29–0.45	0.31–0.42	0.42–0.49
S3	0.43–0.53	0.26–0.38	0.28–0.34	0.43–0.48
S5	0.28–0.41	0.42–0.61	0.38–0.45	0.38–0.43

^a^ Measurement results of Figure 4, mm.

## Data Availability

The study did not report any data and exclude this statement please.

## Supplementary Materials

The following are available online at https://www.mdpi.com/article/10.3390/biomedicines9091138/s1, Figure S1: Surface morphologies and elemental mappings of the porous composite scaffolds.
