# Supplementary material for: Rapid Fabrication of MgNH4PO4·H2O/SrHPO4 Porous Composite Scaffolds with Improved Radiopacity via 3D Printing Process
Source: Biomedicines. 2021 Sep 2;9(9):1138. doi: 10.3390/biomedicines9091138 (PMC8468055; doi:10.3390/biomedicines9091138)
Supplement: Supplementary file 1 [file biomedicines-09-01138-s001.zip › biomedicines-1330391-supplymentary.pdf]

biomedicines

Supporting Information

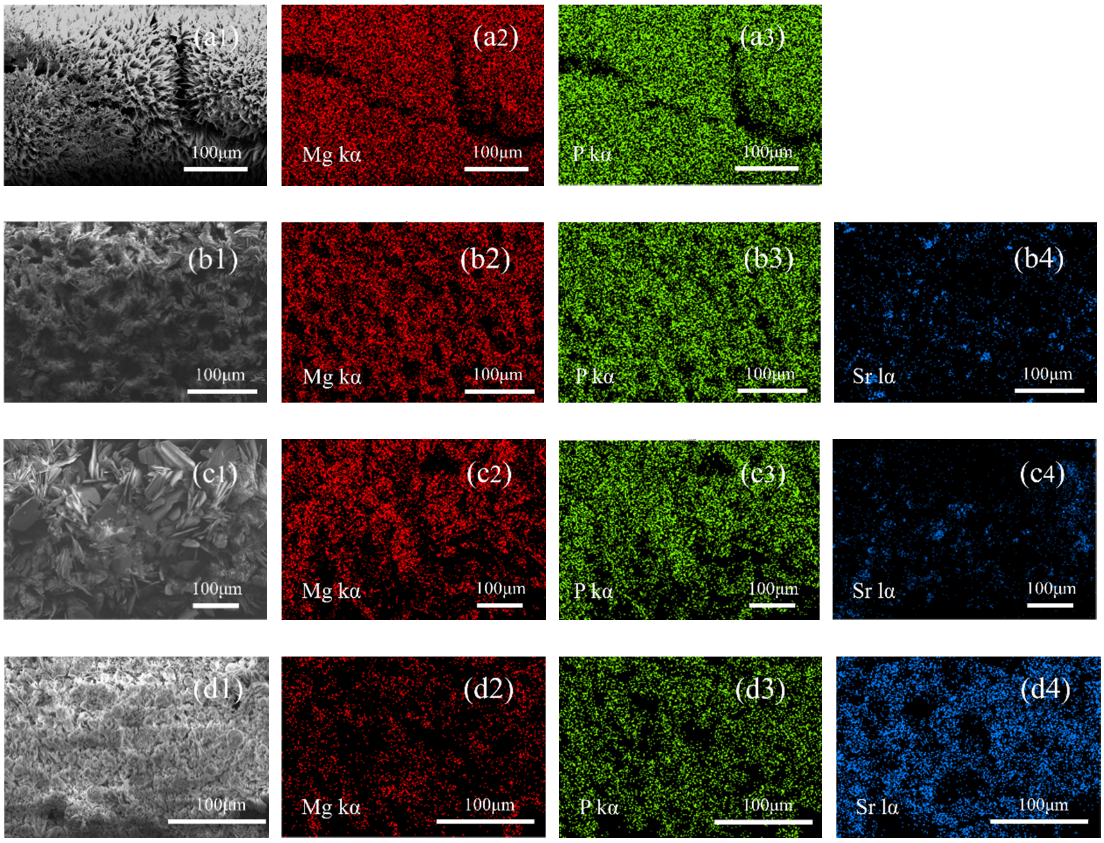

Figure S1. Surface morphologies and elemental mappings of the porous composite scaffolds S0, S1, S3, and S5, (a), (b), (c) and (d), respectively. SEM images (a1, b1, c1, and d1); Mg element (a2, b2, c2, and d2); P element (a3, b3, c3, and d3); and Sr element (b4, c4, and d4).
